# High risk behaviors of injection drug users registered with harm reduction programme in Karachi, Pakistan

**DOI:** 10.1186/1477-7517-4-7

**Published:** 2007-02-10

**Authors:** Arshad Altaf, Sharaf Ali Shah, Najam A Zaidi, Ashraf Memon, Nadeem-ur-Rehman, Norman Wray

**Affiliations:** 1 Community Health Sciences, Aga Khan University, Karachi, Pakistan; 2 Enhanced HIV/AIDS Control Programme, Government of Sindh, Karachi, Pakistan; 3 Department of Medicine, Memorial Hospital, Brown University, USA; 4 United Nations Office for Drug Control and Crime Prevention (UNODC), Islamabad, Pakistan; 5 Marie Adelaide Rehabilitation Center, Karachi, Pakistan

## Abstract

**Background:**

Surveillance data of Sindh AIDS Control Programme, Pakistan suggest that HIV infection is rapidly increasing among IDUs in Karachi and has reached 9% in 2004–5 indicating that the country has progressed from nascent to concentrated level of HIV epidemic. Findings of 2^nd ^generation surveillance in 2004–5 also indicate 104/395 (26.3%) IDUs HIV positive in the city.

**Methods:**

We conducted a cross sectional study among registered IDUs of a needle exchange and harm reduction programme in Karachi, Pakistan. A total of 161 IDUs were included in the study between October–November 2003. A detailed questionnaire was implemented and blood samples were collected for HIV, hepatitis B & C and syphilis. HIV, hepatitis B and C antibody tests were performed using Enzyme Linked Immunosorbent Assay (ELISA) method. Syphilis tests (RPR & TPHA) were performed on Randox kit.

Besides calculating frequencies univariate analysis was performed using *t tests *for continuous variables as age, age at first intercourse and average age of initiation of addiction and *chi square *for categorical variables like paid for sex or not to identify risk factors for hepatitis B and C and syphilis.

**Results:**

Average age of IDU was 35.9 years and average age of initiation of drugs was 15.9 years. Number of drug injections per day was 2.3. Shooting drugs in group sharing syringes was reported by 128 (79.5%) IDUs. Over half 94 (58.3%) reported paying for sex and 64% reported never using a condom. Commercial selling of blood was reported by 44 (28%). 1 of 161 was HIV positive (0.6%). The prevalence of hepatitis B was 12 (7.5%), hepatitis C 151 (94.3%) and syphilis 21 (13.1%). IDUs who were hepatitis C positive were more likely to start sexual activity at an earlier age and had never used condoms. Similarly IDUs who were hepatitis B positive were more likely to belong to a younger age group. Syphilis positive IDUs were more likely to have paid for sex and had never used a condom.

**Conclusion:**

Prudent measures such as access to sterile syringes, rehabilitation and opiate substitution therapies are required to reduce high risk behaviors of IDUs in Pakistan.

## Background

The total number of drug users in Pakistan remains unknown. The National Survey on Drug Abuse in Pakistan in 1993 estimated 2.7 million users of narcotics and psychotropic substances in a total population of 125 million. Heroin, the most prevalent drug of abuse was used by 1.52 million. In this survey injection drug use was first reported in 1.8% addicts in Karachi, a center of commerce, the chief seaport and the largest city of the country with a population of over 10 million [1]. Recent estimates indicate 5 million drug users in Pakistan [2]. Personal communications with field workers, researchers and donors suggest that there is an increasing shift towards injection drug use (IDU) among addicts. Possible reasons for this preference for injection could be the change in heroin quality. The currently available product cannot be inhaled because its impact on lungs is quite severe and has caused respiratory distress in some cases. (Personal Communication, Irshad Khan & Joe Augustine, 2006). There is also limited availability of inhalation quality heroin, and there is a rising cost of other psychotropics [3]. Before the Afghan war the proportion of inhalation addicts was much higher than today and the inhalation material was called "brown sugar" which was heroin however since 2001 "brown sugar" is not available in Karachi and the most common material largely available and used by addicts is called "white stuff" and it can only be injected. (Personal Communication, Irshad Khan & Joe Augustine, 2006).

A study publishing data from Karachi in 1995 reported 25% of a mixed population drug users using injectable drugs and of these 52%, sharing needles. None of the users were tested positive for HIV in that study [4]. However in a different study in Karachi in 1996 out of 242 IDUs, one was HIV positive [5]. In a similar study of IDUs in 2002 no one tested positive for HIV [6].

Global pattern of HIV indicates that injecting drug use has provided a "kick start" to the epidemic [7].

Pakistan has been considered a low prevalence "nascent epidemic" for HIV/AIDS transmission. However, surveillance data from Sindh (Provincial) AIDS Control Programme suggest that Pakistan may already have progressed from low to concentrated level of HIV epidemic since 2003, when 19 IDUs were confirmed HIV positive in Larkana, a small town in the Sindh Province. This was the first such outbreak reported from Pakistan [8].

Present study was conducted in 2003 to assess high risk behaviors and prevalence of HIV, hepatitis B and C and syphilis among registered IDUs of a Needle Exchange Programme developed by Marie Adelaide Rehabilitation Programme. This center was established with support of United Nations Office for Drug Control and Crime Prevention (UNODC) and Joint United Nations Programme on HIV/AIDS (UNAIDS) in 2002. In this research correlates to HIV, HCV, HBV and syphilis were studied.

## Methods

### Study setting

The Marie Adelaide Rehabilitation Programme's drug Rehabilitation Center with the name of "House of Hope" has been providing services to drug addicts in rural Sindh province for the past 20 years. In 2002 a mapping exercise was conducted by UNAIDS and UNODC. An area near Burns Road in Karachi was identified as having a large number of drug addicts. The locality is an urban slum: a densely populated area with limited waste disposal and no city planning. In July 2002 a needle exchange or 'Drop-in Center' was established here with the support of UNAIDS and UNODC. The center had a physician and 10 trained staff, all of them are rehabilitated addicts and with good skills to interact with current addicts. Besides syringe exchange, free condoms, treatment of bacterial sexually transmitted infections (STI), abscess dressing, out patient clinic and bathing the center also provides regular counseling and health education to IDUs. Its working hours are from 7:00 am to 5:00 pm Monday to Saturday and 9:00 am to 1:00 pm on Sundays. A medical doctor provides primary health care during working hours. Outreach workers regularly go in the field to encourage addicts to utilize services of the center. At the time of this study the center was supported by Department for International Development (DFID) through the Futures Group of Europe.

In 2004 a total of 1064 IDUs were registered and 58,145 new syringes were distributed in exchange for 56,846 used syringes. The same year 7815 antiseptic dressings were performed. Number of condoms distributed free of cost was 13,715. Screening for HIV and hepatitis B and C is provided free of cost through the Referral Laboratory of Sindh AIDS Control Programme.

### Study design

We conducted a cross-sectional study at the Marie Adelaide Rehabilitation Center Needle exchange Program (the Drop-In Center) in October–November 2003. We developed a questionnaire and field tested it prior to implementation. Besides information about basic demographic features the questionnaire also inquired about drug use history, daily spending on drugs and their sources of earning, reuse of injection equipment, sharing of injections, number of times treated for addiction, incarceration, sexual behavior, awareness about hepatitis B & C and HIV/AIDS related information.

Although higher sample size does not compensate for the bias that can be introduced through incomplete questionnaires 161 participants were included in the study although UNODC had fixed the sample size as 150. Inclusion criteria was being an IDU and registered with the harm reduction program for at least six months and willing to participate.

Interviewers were health workers and out reach workers of MARC. They were trained on taking the client in confidence, probing for high-risk practices and specially maintaining an unbiased attitude throughout the interview. A verbal consent was taken from each client before starting the interview and collecting the blood sample. The study was reviewed and approved by Ethical Review Board of the Sindh AIDS Control Programme. At the end of interview the client was encouraged to continue to visit the center.

Epi Info 6 and SPSS 10 were used to enter and analyze the data. SPSS 10 was also used to conduct univariate analysis. After the entry was complete the data were carefully cleaned. Serological results were entered on Microsoft Excel sheet.

### Laboratory methods

A phlebotomist of Sindh AIDS Control Programme collected blood samples.

The Referral laboratory of Enhanced HIV/AIDS Control Programme, Government of Sindh performed the following serological tests:

1. HIV antibody test

2. Hepatitis B surface antigen test

3. Hepatitis C antibody test

4. Rapid Plasma Reagin (RPR) test for syphilis

5. Reactive RPR sample were confirmed by TPHA Test

HIV antibody test was performed according to WHO HIV antibody testing strategy guidelines. Initially the HIV antibody test was by the Enzyme Linked Immunosorbent Assay (ELISA) method on Multiscan-MS instrument on Vironostika HIV-Uniform II plus o-Biomeriux kit. Any sample found reactive on initial testing was further tested by another ELISA based assay on different test antigens (on Enzygonist Anti-HIV 1/2 plus-Dade Behring kit) and by the Immunochromatographic method (Determine-ABBOTT Kit). Any sample with an indeterminate result was confirmed on Western Blot or Line immunoassay method. Ten percent of All Non-Reactive samples were rechecked by second an ELISA & Immunochromatographic method as quality control.

Hepatitis B and C tests were performed on Human ELISA kits. RPR (Rapid Plasma Reagin) TPHA tests (Treponema pallidum Hemagglutination Assay) were performed on Randox kit.

## Results

Social and demographic characteristics are explained in Table 1. Majority (60.9%) of IDUs were born in Sindh however all provinces are represented in the studied group. Average age was 35.9 years with a range of 18–63 years. More than half (73.3%) were married. Muslims were predominant (95%). Almost 70% had received no formal education. A majority of study participants (81.3%) were spending the night on streets. The average time on living on the streets was reported to be 7.9 years. The average time of using injection for addiction was 4.4 years. Most common drug in use was heroin followed by combination of Diazepam and Lorazepam (43.7%) and Pheniramine alone (37.8%). Average number of injection for drugs was calculated to be 2.3. Majority (79.5%) IDUs reported shooting drugs in group sharing injection equipment and 8% reported using someone else's used injection equipment 1–2 times in the last 30 days.

**Table 1:** Socio-demographic, injection use and STI/HIV/AIDS behaviors and knowledge of IDUs in Karachi, Pakistan.

**Characteristic**	**Results N = 161 (%)**
Average age	35.9 years (18–63)
**Province of birth**	
Sindh	98 (60.9)
Punjab	40 (24.8)
NWFP	14 (8.7)
Balochistan	7 (4.3)
Outside Pakistan (Afghanistan)	2 (1.2)
**Religion**	
Muslim	151 (95)
Christian	7 (4.4)
Hindu	3 (1.8)
**Education**	
No formal education	111 (68.9)
Education received	47 (29.1) for 7.9 years
Pursuing studies	2 (1.2)
**Married**	
Yes	119 (73.3)^1^
No	42 (26.1)
**Spending the night (last month)**	
At home	30 (18.6)
On streets	131 (81.3)^2^
Average monthly income	Rs. 3512 (US$ 61.6)
Daily spending on addiction	Rs. 102 (US$ 1.7)
**Common types of drugs used in injection (last month)**	
Heroin (White stuff)	157 (97.5)
Diazepam or Lorazepam	70 (43.7)
Meclizine or Promethazine	28 (17.3)
Pheniramine	61 (37.8)
**Addict in near relatives (parents, siblings)**	
Yes	30 (18.6)
No	131 (81.3)
**Average age of initiation of drugs**	
Age of initiation of drugs	15.9 years
Average time initiation of injection	4.4 years
**Injection drug use (last month)**	
Average number of drug injections per day	2.3 {range 1–9 injections)
Previous day's injection with a new syringe	153 (95)
Shooting drugs in group sharing syringes	128 (79.5)
Using anyone else's used syringe one to two times in last 30 days	13 (8)
**Paid for sex (last month)**	
Female	76 (47.2)
Male/Transvestite	3 (1.8)
Boy	15 (9.3)
**Condom use (last month)**	
Never used	104 (64.5)
Sometimes	15 (9.3)
**Sexually transmitted disease symptoms (ever)**	
Urethral discharge	14 (8.6)
Genital ulcers	7 (4.3)
**HIV/AIDS knowledge**	
Heard about HIV/AIDS	149 (92.5)
Transmitted through sharing syringes	116 (72)
Transmitted through unsafe sex	116 (72)
Transmitted through unscreened blood transfusion	17 (10.5)
**Commercial sale of blood (ever)**	
Commercially sold blood	44 (28%)
Average time elapsed between interview and last donation	9.2 months

^1 ^Average number of years of marriage 17.4 years

^2^Average time of living on streets: 7.9 years

### Paying for sex and condom use

Over half of IDUs in Karachi 94 (58.3%) reported paying for sex. Paid sex with a female was reported by 44 (47.2%) IDUs, whereas paid sex with a male or transvestite was reported by three IDUs. Paying for sex with young boys was reported by 9 (9.3%) IDUs. Sixty IDUs (64%) reported never using a condom and eight (9%) informed using a condom sometimes.

### STI/HIV/AIDS

Self reported STI symptoms were reported by 21 (13%) IDUs in Karachi. Majority (92.5%) had heard about AIDS. Seventy two percent informed that HIV transmission is possible by sharing syringes and unsafe sex and 10.5% reported HIV transmission by unsafe blood transfusion.

### Risk factors of IDUs for Hepatitis B & C and Syphilis

Univariate analysis was performed using *t tests *for continuous variables as age, age at first intercourse and average age of initiation of addiction and *chi square *for categorical variables like paid for sex or not to identify risk factors for hepatitis B and C and syphilis (Table 2). IDUs who were hepatitis C seropositive were more likely to start sexual activity at an earlier age and had never used condoms. Similarly IDUs who were hepatitis B positive were more likely to belong to a younger age group. There was difference in the age of initiation of addiction. Syphilis positive IDUs were more likely to have paid for sex and had never used a condom. There was significant difference between the average age of study participants, average age of initiation of drugs and mean age of first sexual contact.

**Table 2:** High risk behaviors for hepatitis B, C and syphilis

**Variable**			
**Risky behavior for hepatitis B**	**Sero positive N (%) mean (SD)**	**Sero negative N (%) mean (SD)**	**P value**

Paid for sex: Yes	6 (60%)	109 (72.7%)	0.38
No	4 (40%)	41 (27.4%)	
Age	28 (8.3)	36.2 (9.5)	0.00
Age of first sexual intercourse	12.7 (7.1)	15.9 (9.7)	0.31
Age of initiation of addiction	14.4 (4.4)	16 (4.7)	0.15
**Risk behavior for hepatitis C**			
Paid for sex: Yes	110 (72.8%)	5 (55.6%)	0.26
No	41 (27.2)	4 (44.4%)	
Age	35.7 (9.7)	36.2 (8.9)	0.88
Age of first sexual intercourse	15.5 (9.7)	18 (8.3)	0.46
Age of initiation of addiction	15.8 (4.8)	15.3 (3.3)	0.75
**Risky behavior for syphilis**			
Paid for sex: Yes	18 (85.7%)	97 (69.8%)	0.13
No	3 (14.3%)	42 (30.2%)	
Age	33.6 (6.2)	36 (10)	0.27
Age of first sexual intercourse	13.3 (9.7)	16 (9.6)	0.22
Age of initiation of addiction	14.1 (2.8)	15.9 (4.8)	0.23

### Serological results

One IDU was HIV positive. The prevalence of hepatitis B was 12 (7.5%), hepatitis C 151 (94.3%) and syphilis 21 (13.1%).

## Discussion

IDUs in Karachi have very high rates of hepatitis C (94%) which has also been documented in other studies [9,10]. Shooting drugs in group sharing syringes is also very high (79.5%). An alarming situation is the commercial sale of blood (28%). The situation is no different from other parts of the world where the HIV seropositivity rates have sharply increased. The 2004 surveillance data of Sindh AIDS Control Programme suggest HIV prevalence to be (332/3736) 9% progressing from nascent to concentrated level of the epidemic [11]. The data collection for the present study finished in December 2003. The surveillance of Sindh (Provincial) AIDS Control Program is continuous.

In a similar study in Lahore [10] IDUs did not have insight into disease transmission. In Karachi the knowledge of IDUs on HIV/AIDS seems adequate (82%). Awareness about hepatitis B & C as a result of sharing needles and syringes is less (60%).

Approximately 50% IDUs reported being in a treatment programme and majority of IDUs wanted to get rid of their addiction habit but could not do so because of unavailability of facilities or could not afford rehabilitation programmes. A high relapse rate could be related to no organized rehabilitation programmes in Pakistan. Discussion with the available rehabilitation programs indicate that as soon as an addict enters any rehabilitation he is made to quit *cold turkey *(drugs stopped immediately). Review of needle exchange programs from Australia, Canada, UK, Netherlands and USA by US General Accounting Office and University of California in 1993 indicate that many programs had a link with proper drug treatment facilities [12-14]. There is no provision of proper detoxification and opiate substitution programmes in the country. There are only a few private and expensive facilities providing detoxification.

Secondly, while the IDUs are in rehabilitation they do not have any opportunity of developing minor skills or regaining their lost skills (if they have any). While in treatment the addicts are involved in prayer, cleaning, cooking, meditation and some recreation along with an afternoon siesta. The addicts coming out of treatment do not have economic opportunities and this may be one of the reasons for relapse. During rehabilitation along with proper detoxification if they could be provided vocational skills such as woodworking, electrical or motor vehicle maintenance, they may have more economic opportunities.

We could not perform logistic regression analysis to truly assess the correlates of infection (especially hepatitis B) because of low power therefore some correlates may be subject to confounding. The multivariable analysis of hepatitis C is too common and HIV too rare while there is no difference for syphilis however, a parsimonious model could be run for hepatitis B. Our study was limited to IDUs from one particular area; however, mapping and integrated behavioral and biological study as part of the 2^nd ^generation surveillance system conducted in the city in December 2004 suggests that this is a very mobile population and will change their spots frequently and will not stay in any particular area for long durations. The study also found 104/395 (26.3%) IDUs selected from different spots of Karachi HIV positive [15].

## Conclusion

The entrance of the HIV virus in this high risk groups requires prudent measures of risk, harm reduction and rehabilitation. Immediate efforts are required for development of proper rehabilitation and opiate substitution programmes. Interventions are also needed to prevent commercial selling of blood.

## Competing interests

The author(s) declare that they have no competing interests.

## Authors' contributions

AA: As the principal investigator he was responsible for study design, data collection, analysis and report writing and developing the manuscript.

SAS: As the programme manager of Provincial AIDS Control Programme and co investigator he worked directly with AA in the field work and reviewing the report and manuscript as well as seeking ethical approval.

NZ: He worked closely with AA in developing and improving the manuscript.

AM: As the pathologist of Provincial AIDS Control Programme he was responsible for blood sample collection and serological testing.

NR: Representing UNODC he supervised the over all study with AA and also reviewed the report and worked on manuscript.

BrNR: He supervises the overall operations of Marie Adelaide Rehabilitation Programme and provided all administrative and field work support in the study. He also reviewed the manuscript.
